# Indications for pediatric lung transplantation in 2025: A new era

**DOI:** 10.1016/j.jhlto.2025.100323

**Published:** 2025-06-18

**Authors:** Nicholas Avdimiretz, Don Hayes, Melinda Solomon, Nicolaus Schwerk, Christian Benden

**Affiliations:** a Division of Pediatric Respiratory Medicine, British Columbia Children’s Hospital and University of British Columbia, Vancouver, British Columbia, Canada; b Division of Pulmonary Medicine, Cincinnati Children’s Hospital Medical Center and University of Cincinnati College of Medicine, Cincinnati, OH; c Division of Pediatric Respirology, The Hospital for Sick Children, University of Toronto, Toronto, Ontario, Canada; d Pediatric Pulmonology and Neonatology, Hannover Medical School, Hannover, Germany; e Division of Pediatric Pulmonology, Boston Children’s Hospital and Harvard Medical School, Boston, MA; f Medical Faculty, University of Zurich, Zurich, Switzerland

**Keywords:** Lung transplant, Pediatric, Indications, Cystic fibrosis, Interstitial lung disease, Pulmonary hypertension

## Abstract

The year 2025 marks an important landmark: almost 40 years since the first pediatric lung transplant (LTX), over 3-5 years since the availability of elexacaftor/tezacaftor/ivacaftor in several countries, and 5-10 years since striking shifts were reported in the diagnoses that accounted for pediatric LTX. We review historic indications for pediatric LTX, highlighting shifts in these over time, and analyze data from the ISHLT International Thoracic Organ Transplant Registry, United Network of Organ Sharing, Canadian Cystic Fibrosis (CF) Registry, and other databases up to the present day. Currently, pediatric CF-related LTX cases are at record lows in many countries. Non-retransplant bronchiolitis obliterans seems to be on the rise as a transplant indication in pediatrics, which is particularly true in the younger age group per ISHLT data. Childhood interstitial lung disease is increasing as an indication, especially in North America. Idiopathic pulmonary arterial hypertension (IPAH) and pulmonary hypertension as a whole now account for record highs as indications for pediatric LTX around the world, with IPAH alone now accounting for nearly 20% of pediatric LTX in the United States, for instance. This information will help guide future international pediatric thoracic transplant consensus guidelines around candidate selection and optimization, placing more emphasis on non-CF considerations.

## Background and Methods

It has now been nearly 40 years since the first lung transplant (LTX) was performed in a patient less than 18 years of age in Canada.^1^ Since that time, there have been significant shifts in the indications for pediatric LTX. Owing to this, currently in the mid-2020s, we are in a new era for pediatric LTX specialists and respiratory medicine providers alike. Here, we examine the current and historic indications for pediatric LTX with focus on shifts in the indications over time by reviewing highly cited literature, including data from the International Society for Heart and Lung Transplantation (ISHLT) International Thoracic Organ Transplant (TTX) Registry and the United Network of Organ Sharing (UNOS) database. The last ISHLT TTX Registry pediatric LTX report is from 2022. We report further data from the Canadian Cystic Fibrosis (CF) Registry, the Eurotransplant Registry, the Scandiatransplant annual data report, and others, although these are limited due to the lack of pediatric-level LTX data reported.^2^ Given that the majority of pediatric LTX in recent years are in North America, and due to limitations in accessing pediatric-specific data on indications from other databases, the focus in analysis will be on United States (US) and Canadian data. Descriptive statistics have been conducted on UNOS data up to January 2025.

## Initial considerations

LTX in infants, children, and adolescents presents unique factors for providers when compared to adults. These factors are important in reviewing the reasons for LTX, since careful consideration of these factors is needed to determine the appropriate indications and timing for LTX. Firstly, the allocation of size-appropriate donor lungs for children results in longer wait times, particularly in less populated areas of the world.^2^, ^3^ Newer organ allocation systems prioritizing children and other strategies implemented in select countries, including deceased donor lobar LTX and living donor lobar LTX, have shown some success.^4^, ^5^

Progression of lung disease in children can be rapid and requires intimate knowledge of the underlying physiology. For example, children with pulmonary vascular disease (PVD)/pulmonary hypertension (PH) may maintain their exercise tolerance despite advanced disease and may progress rapidly to requiring LTX.^6^ Other childhood conditions such as interstitial lung diseases (ILD) present and progress differently depending on specific etiology. As a result, a paucity of pooled data exists on rapidly progressive ILD in children. Further research via international collaborations on managing children with ILD will allow for better classification of indications and timing for pediatric LTX.^7^, ^8^, ^9^

## Transplant volume

The last report from the ISHLT TTX Registry noted 2,323 pediatric deceased donor LTXs from 1992 to 2018, with 907 occurring 2010-2018.^10^, ^11^ About half of pediatric LTXs were performed in North America, 43.6% in Europe, and 10.5% in other countries. Examining the UNOS database from the US up to January 2025, there were 1,512 pediatric LTXs since 1988.^12^ Notably, there were 32 pediatric LTXs in the US in 2020 across all indications, 23 in 2021, and 18 in 2022. The potential effect of the COVID-19 pandemic on this decline is addressed in a previous review.^11^ More recently, there has been an upward trend in volume: 30 in 2023, and 26 in 2024.^12^ Across all age groups <18 years, the most common indication was PH/pulmonary arterial hypertension in 2023 and non-retransplant bronchiolitis obliterans in 2024, which is further outlined below. There has also been a recent rebound in volume in Europe: bilateral LTX (adult and pediatric) for Eurotransplant countries declined from 657 in 2019 to 563 in 2022, now back up to 644 in 2024.^13^ Across Scandiatransplant countries, a similar pattern is seen with 5.2 adult and pediatric LTX per million population in 2019, 4.2 in 2021, and back up to 5.0 in 2024.^14^

## Cystic fibrosis: Historic context

**Figure 1:**
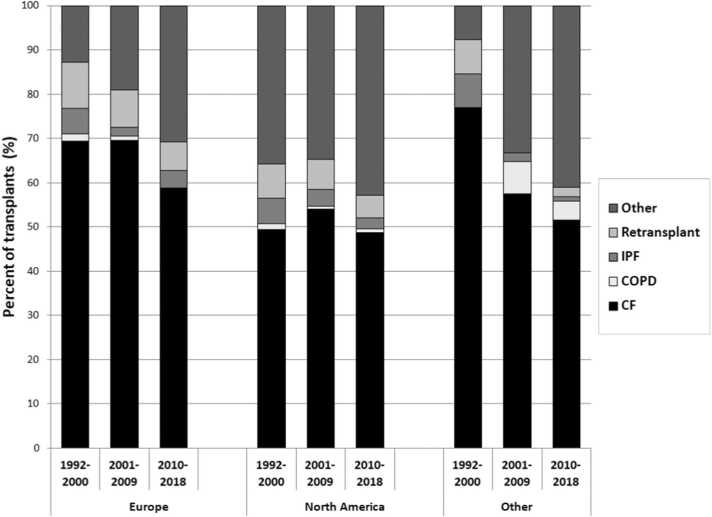
ISHLT International Thoracic Organ Transplant Registry Report 2021. Pediatric lung transplant recipient diagnosis distribution by era and location, 1992-2018. CF, cystic fibrosis; COPD, chronic obstructive pulmonary disease; IPF, idiopathic pulmonary fibrosis; ISHLT, International Society for Heart and Lung Transplantation.

Adapted from Reference.^18^

**Figure 2:**
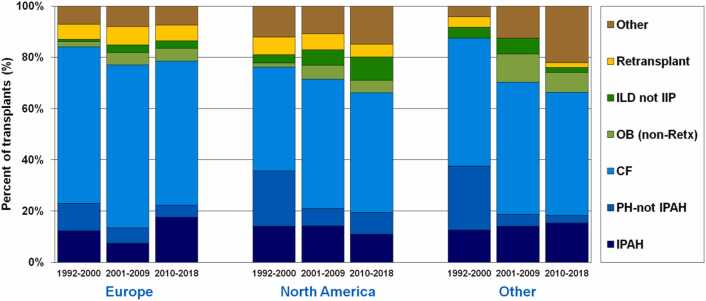
ISHLT International Thoracic Organ Transplant Registry Report 2022. Pediatric lung and heart-lung transplant recipient diagnosis distribution by era and location, 1992-2018. CF, cystic fibrosis; IIP, idiopathic interstitial pneumonia; ILD, interstitial lung disease; IPAH, idiopathic pulmonary arterial hypertension; ISHLT, International Society for Heart and Lung Transplantation; OB, obliterative bronchiolitis; PH, pulmonary hypertension; Retx, retransplant.

Adapted from Reference.^10^

Globally, CF was the leading indication for LTX in patients aged 6-17 years. In the 11-17-year-old age group, CF represented two-thirds of pediatric LTXs.^3^, ^15^, ^16^, ^17^ Until recently, most publications noted CF as being the leading indication for LTX in children and adolescents. A depiction of this data across all indications is shown in Figure 1,^18^ while international data for pediatric LTX and heart-lung transplants (HLTX) is shown in Figure 2.^10^

CF as an indication declined over time in Europe and in non-North American, non-European countries, especially in the most recent era of 2010-2018. A similar trend in CF-related LTX also emerges from UNOS data up to 2025. Pediatric LTXs for CF peaked at 32 in 2013.^11^ CF was the leading indication that year, accounting for 51.6% of pediatric LTXs (previously being as high as 70.2% in 2003). After 2013, however, the proportion of pediatric LTXs for CF declined sharply^11^, ^17^—from a mid-to-late-2010s range of 45%-60% down to 15.6% by 2020.^12^ This shift in indications for pediatric LTX was first noted in 2016, through retrospective analysis of UNOS data (1999-2013), and corroborated by subsequent reviews.^13^, ^18^, ^19^ In Germany, similar trends were seen in adult LTX, with a consistent decline in CF-related LTX occurring since 2018: 58/year in 2012-2019, down to only 10 in 2021.^19^

## Cystic fibrosis: Recent data

**Figure 3:**
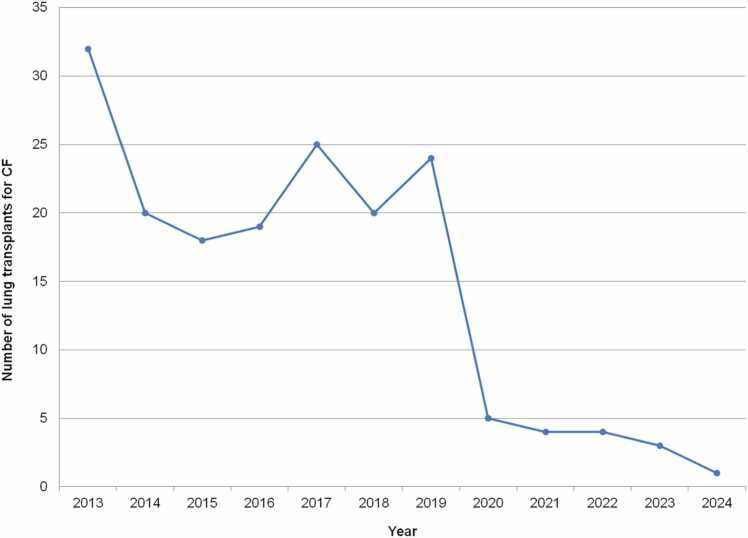
Number of pediatric lung transplants for cystic fibrosis over time from 2013 to 2024 inclusive, United Network of Organ Sharing data. CF, cystic fibrosis.

**Figure 4:**
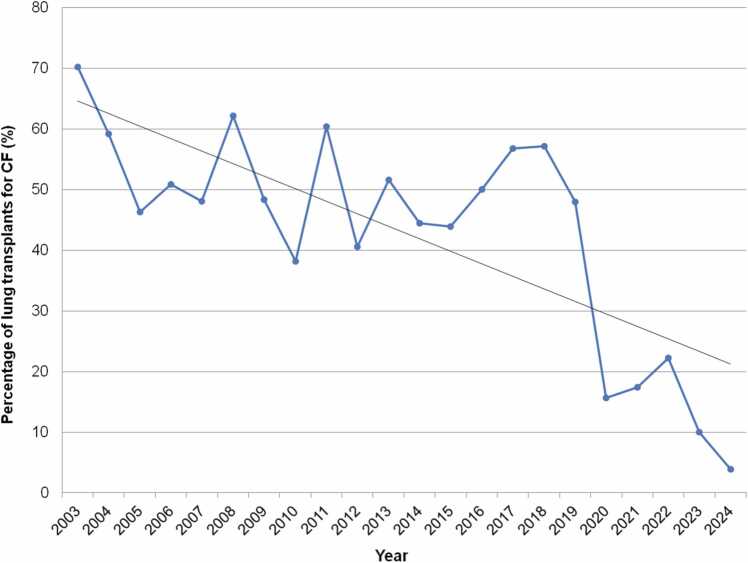
Percentage of total pediatric lung transplants (%) attributed to cystic fibrosis over time from 2003 to 2024 inclusive, United Network of Organ Sharing data. Linear regression trendline in black. Note y axis upper limit is 80%. CF, cystic fibrosis.

Since global data in the ISHLT TTX Registry data is limited, nation-specific data must be used to determine the current state of the indications for pediatric LTX. In the US from January 1988 to January 2025, there have been 709 LTXs done for CF in recipients <18 years of age, accounting for 46.9% during this entire time frame, which is similar to aggregate data since 1992 from the ISHLT TTX Registry. With the introduction of elexacaftor/tezacaftor/ivacaftor (ETI) in the US in 2019 for patients ≥ 12 years of age, there was a subsequent dramatic reduction of pediatric LTXs. Importantly, CF continues to be a rare indication for LTX in children in the US: 4 in 2021, 4 in 2022, and 3 (10% of all transplants, 3/30) in 2023.^20^ Since publication, there has been one in all of 2024. CF accounted for 14.4% of all pediatric LTX in the US from 2020 to 2023, compared to 52.7% in 2016 to 2019. Recently, CF-related pediatric LTXs have again reached *record lows* in the US, at 3.8% of all LTX (1/26) in 2024. Figures follow that depict the absolute number of pediatric LTXs in the US since 2013 (Figure 3) and the percentage of total pediatric LTX attributed to CF since 2003 (Figure 4).

One limitation of this data is that it represents not only selection for LTX based on diagnosis but also transition from the wait list to transplant. However, UNOS data up to 2024 suggests there has been a similar reduction over the years in wait list additions for pediatric LTX candidates with CF, with no significant change in the percent of those wait list candidates being transplanted over time.^21^

The last year (2013) to record over 30 CF-related pediatric LTX in the US occurred around one year after effective CF transmembrane conductance regulator (CFTR) modulator therapies first became available.^11^ The highly effective CFTR modulator therapy (HEMT) ETI is available for patients with at least one F508del allele (approximately 85% of the CF population), and in some countries, for patients with this and one other mutation in the CF gene that is responsive to ETI, resulting in lower need for LTX in those patients in multiple countries.^19^, ^22^ It is important to note that ETI; however, is not accessible in several countries.^23^ Notably, there have been downstream effects to lower rates of pediatric LTX for CF. Less LTX being performed in younger CF patients has led to an aging CF population for those requiring transplantation,^24^ the result of which is to be determined; this could include increased risk of comorbidities such as malignancy, higher BMI, and increased risk of sensitization later in life.^22^

**Figure 5:**
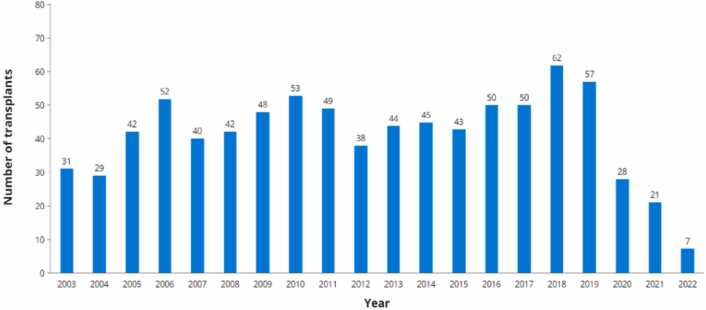
The Canadian Cystic Fibrosis Registry 2022 Annual Data Report. Number of solid organ transplants, almost exclusively for lung transplantation or combined lung with another organ, 2003-2022. Includes pediatric and adult transplants.

Adapted from Reference.^25^

In June 2021, Health Canada approved ETI for use in CF patients ≥ 12 years of age with at least one F508del mutation and further expanded to 6-11-year-olds in 2022. According to the Canadian CF Registry, the median age of individuals with CF has increased over the last two decades to 25.8 years in 2022.^25^ Additionally, the percent predicted FEV_1_ for Canadian CF patients > 6 years has increased significantly by almost 20% in the last 20 years, with ∼70% of children having FEV_1_ over 90% predicted in 2022. Survival has also continued to improve, especially in recent years, with the median age of survival reaching 59.9 years in 2022. The number of CF-related LTX for children and adults in Canada declined dramatically to 7 in 2022, down almost 90% from 2019^25^ (Figure 5). Across all years up to 2022, 14 patients were removed from the LTX wait list. Almost all of these patients were on CFTR modulators.

In recent years, further publications have corroborated these findings. Based on UNOS data linked to the CF Foundation Patient Registry, since 2019 with the release of ETI in the US, there was a significant decline in LTX for adults with CF, resulting in CF-related LTX accounting for only 1.0% of adult LTX in the US in 2022.^26^ Similarly in France, data from the French Agence de la Biomédecine Registry suggested that increased ETI use was a driver in reducing the need for LTX for individuals with CF, with a 55% reduction in LTXs for CF in 2020 compared to previous years; although this was during the COVID-19 pandemic surge, which limited LTX occurrences, there was a continued decline in 2021.^27^ In Germany, pediatric-specific data shows that LTX for CF declined in 2022, which coincided with the approval of ETI use that same year for children over 6 years of age; however, there has been a subsequent increase in CF-related LTX in children in 2024.^13^

## Bronchiolitis obliterans

Unlike CF, bronchiolitis obliterans (BO) is an obstructive lung disease that is rising as an indication for LTX in children. BO involves obstruction of the small airways and is classified based on etiology: post-infectious or post-immune related events, post-hematopoietic stem cell transplant BO syndrome (BOS), or post-LTX BOS (chronic lung allograft dysfunction). Retransplant (repeat LTX) in children for BOS-type chronic lung allograft dysfunction remains rare; however, discussion on this indication is important given that retransplant continues to be associated with lower survival compared to initial LTX.^10^ There has *not* been a significant observable increase in retransplant for children over time. Non-retransplant (non-Retx) BO, which includes all BO types listed above except for repeat LTX, accounted for 9% of pediatric LTX in non-European, non-North American countries, 5% in North America, and 5% in Europe in 2010-2018.^17^ Non-Retx BO was an indication for pediatric LTX in nearly 15% of the 6-10-year-old age group, 8.6% in 1-5-year-olds, and 4.8% in adolescents. Non-Retx BO as an indication is on the rise globally, with more LTXs occurring due to BO in modern eras, especially since 2002 (Figure 2). Non-Retx BO has also been recognized as an increasing indication for younger patients aged 6-10 years in the 2010-2018 era, in terms of numbers and percentage of all LTX.^17^ The reason for this is unclear, whether this relates to a shifting disease process, earlier detection and diagnosis, or earlier referral and/or listing for LTX. In the US, the number of pediatric LTXs occurring for non-Retx BO has remained low for many years, averaging 2-3/year since 2013. However, there has been a recent spike in pediatric LTX for non-Retx BO in 2024 at 7 cases.^21^ Unfortunately, the underlying etiology of non-Retx BO is not recorded. We hypothesize this may be due to (1) increased frequency or severity of BO following the height of the COVID-19 pandemic, since BOS has been reported after COVID-19 infection,^28^ (2) increasing numbers of patients undergoing hematopoietic stem cell transplan, and/or (3) an ongoing shift towards younger patients being transplanted for non-Retx BO, as was noted in the 2019 ISHLT TTX Registry report. This is an area that needs continued evaluation and investigation.

## Interstitial lung diseases

Childhood interstitial lung disease (chILD) is on the rise as an indication for pediatric LTX, at least in the US.^11^, ^20^ These are heterogeneous disorders, with diagnostic criteria established by respiratory and chILD societies,^8^, ^29^ which include but are not limited to surfactant protein (SP) disorders, lung growth abnormalities (including alveolar capillary dysplasia with misalignment of pulmonary veins), pulmonary fibrosis, interstitial pneumonias, and entities being increasingly recognized like chILD associated with STAT3 or COPA (coatomer protein complex subunit alpha) mutations. SP disorders may result in alveolar proteinosis and include surfactant protein B deficiency (SP-B), surfactant protein C deficiency (SP-C), and mutation in the genes encoding adenosine triphosphate binding cassette protein member A3 (ABCA3) and thyroid transcription factor (NKX2.1).

Worldwide across all ages, ILD accounted for at least 11.5% (193/1,677) of all pediatric LTX between 2002 and 2018.^17^ This includes categories labeled as “ILD”, “ILD-Other Cause”, all disorders of surfactant dysfunction (such as SP-B, SP-C, and ABCA3), and bronchopulmonary dysplasia (BPD). These percentages do not include non-Retx BO or unknown diagnoses. The distribution of these indications is similar around the world. In terms of age distribution, SP-B, SP-C, and ABCA3 diagnoses accounted for a large proportion of pediatric LTX recipients < 1-year-old (32.3% of that age group); whereas “ILD” and “ILD-Other Cause” labels were fairly consistent across age groups but were highest in < 1-year-olds (17.8% of that age group).^17^ LTX for BPD is rare, although a small subset of infants with severe BPD have progressive respiratory failure despite maximal medical therapies and may be LTX candidates at certain centers. In more modern eras (2010-2018), more patients with chILD are undergoing LTX in North America (Figure 2). Additionally, chILD patients undergoing LTX are now older, with an increasing proportion of LTXs for chILD in the 11-17-year-old age group.^17^

**Figure 6:**
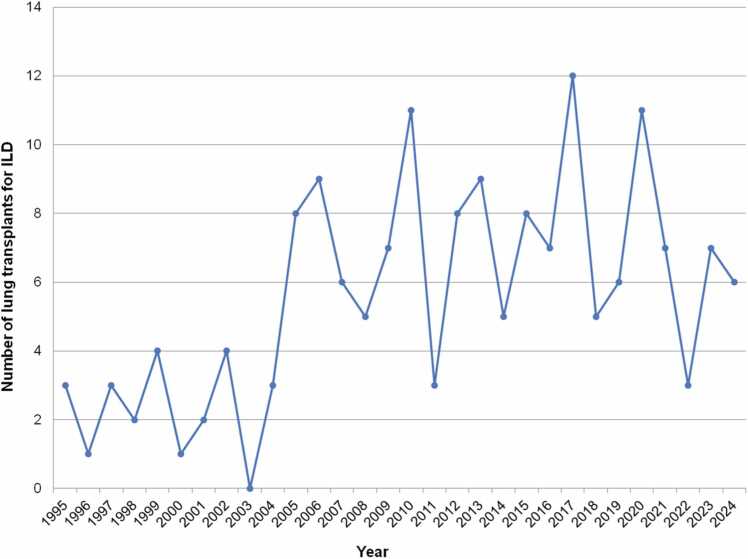
Number of pediatric lung transplants for interstitial lung disease over time from 1995 to 2024 inclusive, United Network of Organ Sharing data. ILD, interstitial lung disease.

**Figure 7:**
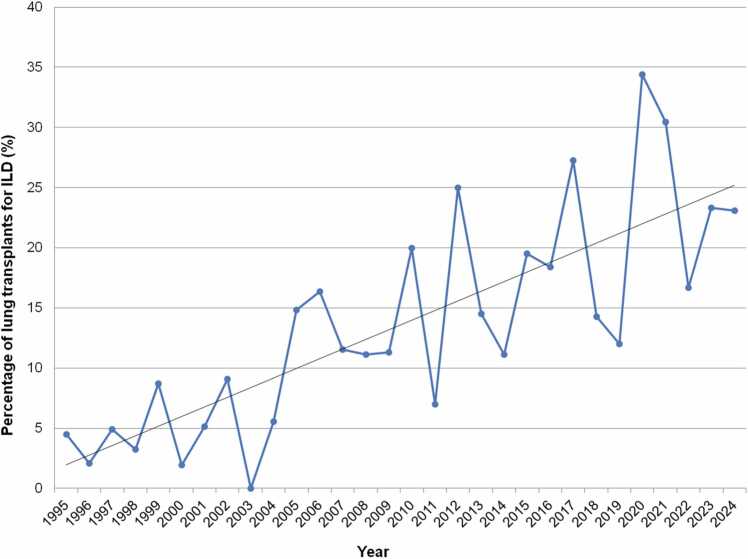
Percentage of total pediatric lung transplants (%) attributed to interstitial lung disease over time from 1995 to 2024 inclusive, United Network of Organ Sharing data. Linear regression trendline in black. Note y axis upper limit is 40%. ILD, interstitial lung disease.

Previous analysis of UNOS data reported that chILD-related LTX has been increasing in the US for decades, both in terms of numbers and in terms of percentage of LTX performed.^11^, ^20^ Recently, investigators reported that LTX for chILD averaged 32.4% of all LTX in 2020-2023, up from 20.3% in 2016-2019; however, this analysis included BO.^20^ In this manuscript, we have removed BO from the chILD analysis, since BO is analyzed separately. In this US, there were 6 pediatric LTXs for chILD in 2024, 7 in 2023, and 3 in 2022. Analyzing UNOS data using 5-year intervals from 1995 to 2024, new data shows: chILD reached *record highs* in the most recent era as an indication for LTX in terms of percentage. The diagnoses accounted for a high of 26.4% in 2020-2024 inclusive, versus 18.3% in 2015-2019, 15.2% in 2010-2014, 13.1% in 2005-2009, and 4.2% in 2000-2004—demonstrating a steady increase for chILD as a pediatric LTX indication over time. Annual number of pediatric LTXs in the US for chILD is depicted (Figure 6), as well as the increasing percentage attributed to chILD (Figure 7). Note that no other databases were available for analysis on chILD-related LTX. Potential factors resulting in increasing chILD-related LTX include improved accuracy in diagnosis and awareness of these rare conditions, improved familiarity on optimal timing of referral and listing, and/or improved bridging strategies for more critically ill patients.

## Pulmonary vascular diseases

PVDs, including idiopathic pulmonary arterial hypertension (IPAH), are important and increasing reasons for referral and selection of a child for LTX.^3^, ^11^, ^17^, ^20^ Historically, IPAH has been the most common indication for pediatric LTX recipients aged 1-5 years, internationally accounting for 26.7% in that age group.^17^ Globally, non-IPAH PH has been recorded as the second most common indication for LTX in infants next to chILD, accounting for 25.8% of LTX in that age group. IPAH accounted for 15.9% (63/397) of pediatric LTX in Europe in the most recent ISHLT-reported era, compared to 8.9% (37/417) in North America and 13% (13/100) in other countries.^17^ Smaller proportions were noted for non-IPAH PH, with the leading region being North America, reporting 7.9% (33/417). The most recently published ISHLT TTX Registry Report focused on PVDs and described an increasing number of LTX and HLTX recipients with these diagnoses, particularly IPAH, when comparing 2001-2009 (17.9%) and 2010-2018 (20.5%).^10^ In addition, shifting practice toward LTX and away from combined HLTX in children with PVDs is reflected in international data.

**Figure 8:**
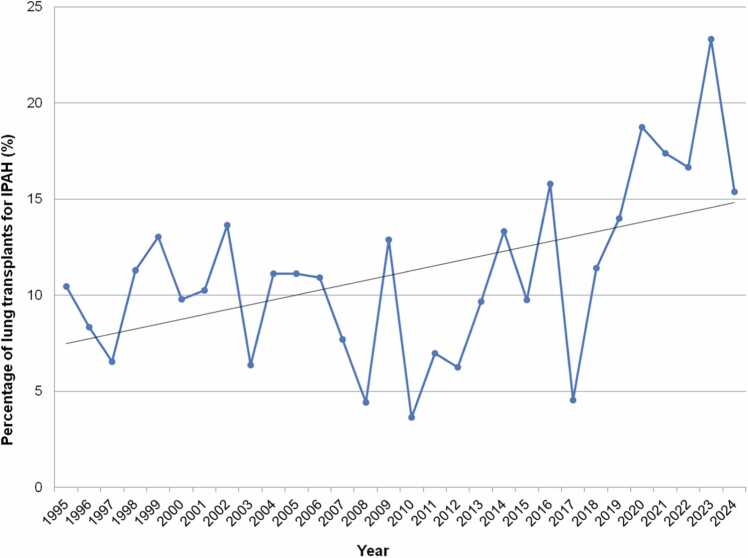
Percentage of total pediatric lung transplants (%) attributed to idiopathic pulmonary arterial hypertension over time from 1995 to 2024 inclusive, United Network of Organ Sharing data. Linear regression trendline in black. Note y axis upper limit is 25%. IPAH, idiopathic pulmonary arterial hypertension.

**Figure 9:**
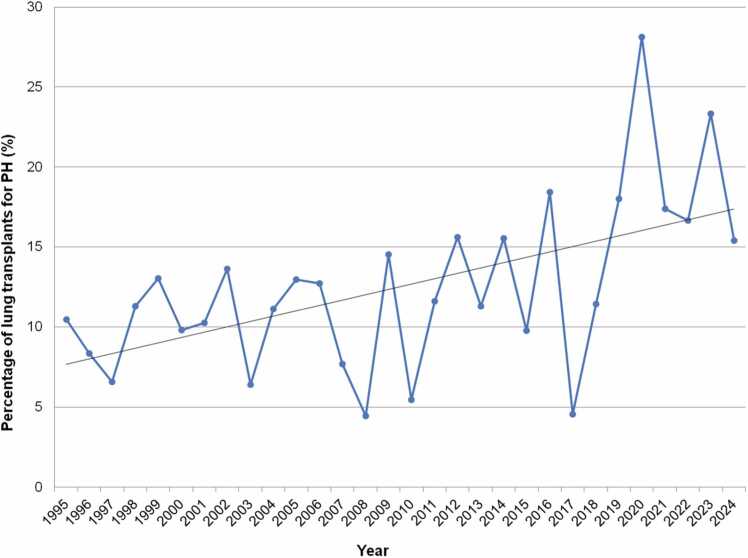
Percentage of total pediatric lung transplants (%) attributed to pulmonary hypertension (both primary (IPAH) and secondary) over time from 1995 to 2024 inclusive, United Network of Organ Sharing data. Linear regression trendline in black. Note y axis upper limit is 30%. IPAH, idiopathic pulmonary arterial hypertension; PH, pulmonary hypertension.

Reviewing UNOS data up to 2025, similar trends are seen. An increase in the proportion of LTXs occurring for both IPAH and for PH altogether (primary and secondary) was observed over time, especially from 2014 onward.^11^, ^20^ Now, while there is no clear increase (or decline) in the absolute number of LTX cases occurring for IPAH or non-IPAH PH, there has been a continued increase in the percentage that IPAH and PH represent as indications for pediatric LTX, particularly from 2020 to 2024.^12^ Pediatric LTX for IPAH is now at an all-time high; IPAH alone represents 18.6% of cases in the US in 2020-2024, compared to 11.0% in 2015-2019 and 8.0% in 2010-2014 (Figure 8). Similarly, PH as a whole (primary and secondary, although not including Eisenmenger syndrome since separately categorized per UNOS) has also increased to all-time highs as an indication for LTX. This now accounts for 20.9% of LTXs in recent years, compared to 12.5% in the previous era (Figure 9).

While these trends are not surprising given the simultaneous decline of CF-related LTX, the increase seen for IPAH and PH over the past 3-5 years is still significant. It could also be argued that the increase in LTXs for these conditions may be related to a reduction in combined HLTXs. In fact, there has been a decline since 2002 in IPAH as an indication for pediatric HLTX in recent years in the US.^11^ Overall, pediatric HLTX remains rare.

## Conclusions

Several important conclusions can be made from this data across these indications for LTX in children and adolescents. At present in 2025, CF is a rare indication for pediatric LTX in the US, Canada, and other countries where ETI is more widely available. HEMTs have widely become the standard of care and are driving demographic changes in pediatric LTX, as corroborated by ISHLT TTX Registry data and reports from various countries, including France and Germany. Non-Retx BO, while still not accounting for a significant number of pediatric LTXs, appears to be on the rise as an indication for pediatric LTX. ChILD is an increasing subgroup of pediatric LTX recipients, especially in North America, and represents an aging group over time. Finally, there has been an increase internationally in the proportion of children undergoing LTX for IPAH and PH. Most striking is that, based on 2020-2025 data, IPAH now accounts for an all-time high of 18.6% of pediatric LTXs in the US. As CF is now a rare indication for pediatric LTX, we recommend individualized referral and listing criteria for these children, as these patients may have CFTR variants not conducive to HEMT or may be non-responders to these therapies. Moreover, the indications shifting away from CF described here and toward chILD and PVDs should help guide the pediatric thoracic transplant community in their approach to referring and selecting children as LTX candidates.

## Declaration of Competing Interest

The authors declare that they have no known competing financial interests or personal relationships that could have appeared to influence the work reported in this paper.
